# Early Season Pediatric Influenza B/Victoria Virus Infections Associated with a Recently Emerged Virus Subclade — Louisiana, 2019

**DOI:** 10.15585/mmwr.mm6902e1

**Published:** 2020-01-17

**Authors:** Daniel Owusu, Julie Hand, Mark W. Tenforde, Leora R. Feldstein, Juliana DaSilva, John Barnes, Grace Lee, Juliet Tran, Theresa Sokol, Alicia M. Fry, Lynnette Brammer, Melissa A. Rolfes

**Affiliations:** ^1^Epidemic Intelligence Service, CDC; ^2^Influenza Division, National Center for Immunization and Respiratory Diseases, CDC; ^3^Louisiana Department of Health; ^4^Tulane University Preventive Medicine Residency, New Orleans, Louisiana.

Multiple genetically distinct influenza B/Victoria lineage viruses have cocirculated in the United States recently, circulating sporadically during the 2018–19 season and more frequently early during the 2019–20 season (*1*). The beginning of the 2019–20 influenza season in Louisiana was unusually early and intense, with infections primarily caused by influenza B/Victoria lineage viruses. One large pediatric health care facility in New Orleans (facility A) reported 1,268 laboratory-confirmed influenza B virus infections, including 23 hospitalizations from July 31 to November 21, 2019, a time when influenza activity is typically low. During this period, Louisiana also reported one pediatric death associated with influenza B virus infection. An investigation of the influenza B virus infections in Louisiana, including medical and vaccine record abstraction on 198 patients, primarily from facility A, with sporadic cases from other facilities in the state, found that none of the patients had received 2019–20 seasonal influenza vaccine, in part because influenza activity began before influenza vaccination typically occurs. Among 83 influenza B viruses sequenced from 198 patients in Louisiana, 81 (98%) belonged to the recently emerged B/Victoria V1A.3 genetic subclade. Nationally, to date, B/Victoria viruses are the most commonly reported influenza viruses among persons aged <25 years (*2*). Of the 198 patients in the investigation, 95% were aged <18 years. Although most illnesses were uncomplicated, the number of hospitalizations, clinical complications, and the reported pediatric death in Louisiana serve as a reminder that, even though influenza B viruses are less common than influenza A viruses in most seasons, influenza B virus infection can be severe in children. All persons aged ≥6 months should receive an annual influenza vaccination if they have not already received it (*3*). Antiviral treatment of influenza is recommended as soon as possible for all hospitalized patients and for outpatients at high risk for influenza complications (including children aged <2 years and persons with underlying medical conditions) (*4*).

In November 2019, a field investigation was conducted to characterize the early influenza B virus–associated illnesses in Louisiana and to determine the influenza B virus subclades responsible for the outbreak. Medical chart abstraction, using a standard case report form, was conducted for 198 persons with laboratory-confirmed influenza B virus infection who had respiratory specimens submitted to the Louisiana Public Health Laboratory, including 173 outpatients and 25 hospitalized patients, from May 24 to November 21, 2019. Among 198 completed medical chart abstractions, 181 patients (158 outpatients and 23 inpatients) were from facility A; 17 were from other facilities in Louisiana.

The percentage of health care visits for influenza-like illness in Louisiana began to increase in mid-August 2019, corresponding to surveillance week 33 (Figure). Illness onset among the 198 patients occurred during May 24–October 29, 2019 with median onset during surveillance week 38 (ending September 21, 2019). The median age of patients was 6 years (range = <1 month–29 years); 95% were aged <18 years, reflecting both the increased circulation of influenza B viruses in children and the general patient population of facility A. None of the 198 patients had received the 2019–20 seasonal influenza vaccine before becoming ill, likely at least in part because influenza activity began early, before influenza vaccine campaigns start. Most patients reported subjective fever (95%), cough (68%), and runny nose (61%). Among the 173 outpatients, 41 (24%) had an underlying medical condition, the most common of which was asthma (Table); 17 (10%) had a complication associated with their infection, and 122 (71%) were prescribed influenza antivirals. Among 25 hospitalized patients, 14 (56%) had an underlying medical condition, 23 (92%) were prescribed influenza antivirals, 11 (44%) had complications associated with their infection, and six (24%) were admitted to intensive care units.

**FIGURE:**
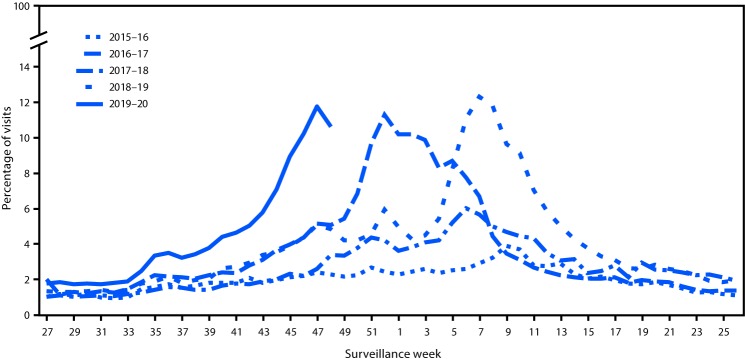
Percentage of visits for influenza-like illness* reported by sentinel clinics, by surveillance week — Louisiana, influenza seasons 2015–16 to 2019–20 * Defined as fever (temperature of ≥100°F [≥37.8°C], oral or equivalent) and cough or sore throat, without a known cause other than influenza.

**TABLE:** Underlying medical conditions and influenza-associated complications in patients with influenza B virus infections (N = 198) — Louisiana, 2019

Characteristic	No. (%)	
	Outpatients (n = 173)	Inpatients (n = 25)
**Prescribed influenza antivirals**	122 (71)	23 (92)
Underlying medical conditions*		
Asthma	28 (16)	9 (36)
Cardiovascular disease	0	2 (8)
Febrile seizure	3 (2)	0
Blood disorder	4 (2)	3 (12)
Immunosuppression	0	1 (4)
Neurologic disorder	6 (3)	2 (8)
Neuromuscular disorder	0	2 (8)
Premature birth	3 (2)	0
**Complications^†^**		
Acute otitis media	10 (6)	0
Acute respiratory failure	0	2 (8)
Asthma exacerbation	4 (2)	4 (16)
Myopericarditis	0	1 (4)
Pneumonia	5 (3)	3 (12)
Rhabdomyolysis	0	1 (4)
Seizures	1 (0.6)	0
Sepsis	0	3 (12)

* Some patients had more than one underlying medical condition.

^†^ Some patients had more than one complication.

Among 83 influenza B viruses sequenced from the 198 patients, 81 (98%) belonged to the influenza B/Victoria V1A.3 subclade, which began circulating in the United States in the latter half of the 2018–19 influenza season (*5*). One of the detected viruses in Louisiana belonged to subclade V1A.1, which is the subclade of the influenza B/Victoria component (B/Colorado/06/2017) of the 2019–20 Northern Hemisphere vaccine. One of the 83 viruses could not be classified.

## Discussion

Typically, influenza B viruses circulate during the spring, near the end of influenza season; however, in the current 2019–20 season, influenza B/Victoria viruses are the predominant circulating influenza virus in the United States to date (*2*). Influenza B viruses have not been the predominant virus in the United States since the 1992–93 season (*6*). B/Victoria viruses did not circulate widely during the past three influenza seasons, accounting for <10% of influenza virus isolates reported during the 2016–17 to 2018–19 seasons.* Of the multiple genetically distinct B/Victoria virus subclades, viruses with two amino acid deletions in the hemagglutinin protein, belonging to the V1A.1 subclade, and viruses with three amino acid deletions, belonging to the V1A.2 or V1A.3 subclades, cocirculated during May–September, 2019 (*1*). Although the V1A.1 and V1A.3 subclades are genetically distinct, sera from previous studies conducted among humans vaccinated with a V1A.1 virus cross-reacted well with B/Victoria viruses with a three amino acid deletion, such as the V1A.3 viruses (*1*). These findings suggest that vaccination with the current season’s vaccine might offer protection against circulating B/Victoria viruses.

Nationally, from September 29 to December 28, 2019, influenza B viruses accounted for 59.2% of influenza-positive results reported by public health laboratories, and, among those with lineage testing, 97.9% belonged to the B/Victoria lineage (*2*). Through December 28, B/Victoria viruses were the most commonly reported influenza viruses among persons aged <25 years, and influenza A(H1N1)pdm09 viruses were the most commonly reported among persons aged ≥25 years (*2*). In addition, 70% of influenza-associated hospitalizations among children reported through the Influenza Hospitalization Surveillance Network (Shikha Garg, personal communication, January 2020) and 18 of 27 influenza-associated pediatric deaths were associated with influenza B viruses (five of the 18 deaths had virus lineage reported and all were B/Victoria) (*2*).

Symptoms and outcomes among patients with influenza B/Victoria virus infection in Louisiana were typical of seasonal influenza A or B virus infections (*7*,*8*), primarily resulting in uncomplicated respiratory illness. However, the number of hospitalizations, clinical complications, and the reported pediatric death in Louisiana serve as a reminder that, even though influenza B viruses are less common than influenza A viruses in most seasons, influenza B virus infection can be severe in children. Common complications of influenza, such as pneumonia and bacterial coinfection, have previously been as frequent among children hospitalized with influenza B virus infection as among those with influenza A virus infection (*8*). During 2010–2016, the percentage of influenza B viruses detected in children who died with influenza was higher than the percentage of B viruses detected in the general pediatric population (*9*). Further, a large autopsy series found that the histology of fatal influenza B virus infection was similar to that of fatal influenza A virus infection; however, younger patients who died with influenza B virus infection were less likely to have bacterial coinfection and frequently had myocardial injury (*10*).

Influenza activity is expected to continue for many weeks in the United States; additional hospitalizations and deaths, including among children, are expected to occur. To prevent influenza, all persons aged ≥6 months should receive an annual influenza vaccine, and it is not too late to be vaccinated for the 2019–20 season (*3*). In addition, influenza antiviral treatment is an important tool to reduce symptom duration and the risk for complications and is recommended as soon as possible for all influenza patients who are hospitalized and outpatients at high risk for influenza-associated complications, including children aged <2 years and those with underlying medical conditions^†^ (*4*). Resources, such as HealthMap Vaccine Finder (https://www.vaccinefinder.org) and Medfinder (https://www.medfinder.org), are available to assist in identifying places to get age-appropriate influenza vaccines or fill prescriptions for influenza antivirals.

**Summary**

**What is already known about this topic?**

Influenza B viruses have not predominated in the United States for 27 years. Influenza B virus infection is more common among children and can cause complications, resulting in hospitalization or death.

**What is added by this report?**

Early influenza B/Victoria virus activity in Louisiana resulted in illnesses in children that were similar to typical seasonal influenza; however, some illnesses were severe, and one death was reported.

**What are the implications for public health practice?**

Annual influenza vaccination is recommended for all persons aged ≥6 months. It is not too late to be vaccinated for the 2019–20 influenza season. Influenza antiviral treatment is recommended for those hospitalized with influenza or outpatients with influenza who are at risk for complications.

## Footnotes

* https://gis.cdc.gov/grasp/fluview/fluportaldashboard.html.

† https://www.cdc.gov/flu/professionals/antivirals/summary-clinicians.htm.
